# Pregabalin Depresses Cerebellar Parallel Fiber–Purkinje Cell Synaptic Transmission by Modulating Glun2a-Containing Nmda Receptors in Mice In Vitro

**DOI:** 10.3390/ijms27114660

**Published:** 2026-05-22

**Authors:** Mei-Rui Li, Xu-Dong Zhang, Li Chen, Yi-Dan Zhang, Chun-Yan Wang, Zi-Yu Zhao, Mo Zhou, Chun-Ping Chu, De-Lai Qiu

**Affiliations:** 1 Department of Physiology and Pathophysiology, College of Medicine, Yanbian University, Yanji 133002, Chinazxd18804312627@163.com (X.-D.Z.); cl13244221355@163.com (L.C.); zhangyidan0222@163.com (Y.-D.Z.); 2 Institute of Brain Science, Jilin Medical University, Jilin 132013, China; happywcy@163.com (C.-Y.W.); 15543472863@163.com (M.Z.); 3 Department of Physiology, College of Basic Medicine, Jilin Medical University, Jilin 132013, China

**Keywords:** pregabalin, cerebellar parallel fiber, Purkinje cell, excitatory postsynaptic currents (EPSCs), gluN2A-containing NMDA receptor, miniature excitatory postsynaptic currents (mEPSCs), protein kinase A (PKA)

## Abstract

Pregabalin (PGB) exerts its therapeutic effects by binding to the α_2_δ auxiliary subunits of voltage-gated calcium channels and modulates synaptic transmission in the brain. However, its influence on cerebellar parallel fiber–Purkinje cell (PF–PC) synaptic transmission remains unclear. In the present study, we investigated the effects of PGB on PF–PC synaptic transmission using whole-cell patch-clamp recording, glutamate fluorescence imaging, immunohistochemistry, co-immunoprecipitation, Western blotting, and pharmacological approaches. Micro-application of PGB to the cerebellar molecular layer induced a concentration-dependent inhibition of PF–PC excitatory postsynaptic currents (EPSCs), accompanied by an increased paired-pulse ratio. The inhibitory effect of PGB on PF–PC EPSCs was abolished by extracellular blockade of N-methyl-D-aspartate receptors (NMDAR) or their GluN2A subtype, as well as by disruption of α_2_δ-1–NMDAR complexes, but not by intracellular NMDAR inhibition. Glutamate sensor imaging further showed that PGB markedly reduced the fluorescence intensity of glutamate release evoked by PF stimulation. In the presence of tetrodotoxin (TTX) and a gamma-aminobutyric acid type A (GABAA) receptor antagonist, PGB reduced the frequency of miniature excitatory postsynaptic currents (mEPSCs) without affecting their amplitude. The PGB-induced reduction in mEPSC frequency was fully abolished by extracellular blockade of GluN2A-containing NMDARs or disruption of α_2_δ-1–NMDAR complexes. Similarly, the inhibitory effects of PGB on PF–PC EPSCs and mEPSCs were eliminated by extracellular PKA inhibition, but not by intracellular protein kinase A (PKA) inhibition. Western blot analysis showed that PGB significantly increased PKA phosphorylation in the molecular layer of the cerebellar cortex. Immunoreactivity for GluN2A and α_2_δ-1 subunits was colocalized within the molecular layer and abundantly distributed around the dendrites and somata of PCs. Co-immunoprecipitation further verified that α_2_δ-1 was co-precipitated with GluN1 in cerebellar molecular layer tissue samples. The results indicate that PGB depresses glutamate release from parallel-fiber terminals in the mouse cerebellar cortex through the presynaptic α_2_δ-1-coupled GluN2A-containing NMDAR/PKA signaling pathway, thereby attenuating PF–PC synaptic transmission.

## 1. Introduction

Pregabalin (PGB) is the pharmacologically active S-enantiomer of the racemic 3-isobutyl γ-aminobutyric acid (GABA) analog. It exhibits anticonvulsant, anxiolytic, sedative, antidepressant, and analgesic properties, and is thus widely prescribed as a first-line therapeutic agent in clinical practice. Although PGB is structurally a GABA analog, it does not directly bind to GABAA, GABAB, or benzodiazepine receptors [1,2,3]. Instead, PGB binds to the α_2_δ auxiliary subunits of voltage-gated calcium channels (VGCCs) and modulates multiple forms of synaptic transmission by regulating the release of major neurotransmitters, including glutamate, GABA, and norepinephrine [4,5,6,7]. α2δ subunits regulate the cell-surface expression and biophysical properties of VGCCs by modulating anterograde trafficking and endocytosis. Accordingly, they facilitate the synaptic accumulation of VGCCs [8], enhance presynaptic neurotransmitter release [9], promote active zone maturation [10], and regulate synaptic plasticity [10,11,12]. Notably, α2δ subunits are abundantly expressed in the cerebellar cortex. These subunits modulate climbing fiber–Purkinje cell (CF–PC) synaptic transmission by suppressing presynaptic neurotransmitter release, thereby refining functional synaptic organization.

PGB exhibits a higher affinity for the α_2_δ-1 isoform than for α_2_δ-2 in multiple brain regions, including the cerebral cortex, hippocampus, and cerebellum [5,13,14]. PGB modulates the trafficking of presynaptic VGCCs to the neuronal membrane [15,16,17,18], as well as protein synthesis, signal transduction, and the activity of multiple ion channels through intracellular signaling pathways [15,16,17,18]. PGB binds to the α_2_δ-1 subunit and strengthens the interaction between BK channels and Cav2 channels [19]; it also suppresses the activation of ATP-sensitive potassium channels, thereby reducing neuronal excitability in dorsal horn neurons of the rat spinal cord [20]. Furthermore, activation of the α_2_δ-1 subunit potentiates NMDA receptor activity, facilitates NMDA receptor-mediated glutamatergic synaptic transmission, and consequently increases excitatory glutamatergic input to dorsal root ganglion neurons [21].

It has been demonstrated that the α_2_δ-1 subunit can couple with NMDA receptors to form functional complexes in the cell membrane of rodents and humans. These complexes play critical roles in the development of neuropathic pain and the therapeutic effects of gabapentin [22,23]. Previous studies have also confirmed that α_2_δ-1 associates with NMDA receptors to form such complexes, thereby modulating NMDA receptor channel activity [24,25]. Specifically, the α_2_δ-1 subunit interacts with NMDA receptors through its C-terminus to enhance the functional activity of NMDA receptors [6]. Given that PGB has a high affinity for the α_2_δ-1 subunit, PGB binding to the α_2_δ-1–NMDA receptor complex inhibits the activity of the complex and thereby suppresses NMDA channel function [25]. This action reduces D-serine levels, lowers the current ratio of NMDA to non-NMDA receptors, and shortens the decay time of NMDA receptor-mediated EPSCs [26,27].

The cerebellar cortex is primarily composed of PCs, molecular layer interneurons, granule cells, and Golgi cells. Cerebellar PCs receive two major excitatory inputs from PFs and climbing fibers, and serve as the sole output of the cerebellar cortex to the deep cerebellar nuclei, regulating essential physiological functions including motor planning, motor execution, and movement coordination [28]. PFs are the axons of granule cells; they receive input from mossy fibers and form excitatory synapses with PCs and molecular layer interneurons. Signaling along the mossy fiber–granule cell pathway relies predominantly on glutamate release from PFs, which drives excitatory synaptic transmission and plasticity at PF–PC and PF–molecular layer interneuron synapses. PF–PC synaptic transmission and plasticity are critical for the regulation of fine motor control and motor learning [28,29].

VGCCs containing α_2_δ subunits are widely distributed throughout the cerebellar cortex and include P/Q-, N-, and R-type calcium channels. Among these subtypes, N-type calcium channels are predominantly expressed in dendrites, whereas P/Q-type and R-type channels are present in both the dendrites and somata of PCs. These observations indicate that α_2_δ subunits exert an important regulatory influence on VGCC activity within the cerebellum [30,31]. α_2_δ-1 subunit-containing VGCCs are expressed in cerebellar granule cells and participate in the modulation of glutamatergic neurotransmission [32]. In contrast, the α_2_δ-2 isoform modulates climbing fiber–Purkinje cell synaptic transmission by attenuating presynaptic neurotransmitter release, thereby shaping structural and functional synaptic organization in the cerebellum [33,34]. Importantly, PGB exhibits a markedly higher affinity for α_2_δ-1 subunit-containing VGCCs in the brain compared with the α_2_δ-2 isoform. This suggests that PGB may exert diverse modulatory effects on cerebellar circuits via regulation of α_2_δ-1-containing VGCCs [35], and may contribute to PGB-induced cerebellar dysfunction and motor disorders such as ataxia [30,36]. Collectively, α_2_δ-1 subunit-containing VGCCs are expressed in cerebellar granule cells and display high affinity for PGB, implying that PGB modulates PF–PC synaptic transmission in the cerebellum. Accordingly, the present study aimed to elucidate the molecular mechanisms by which PGB regulates PF–PC synaptic transmission in the mouse cerebellar cortex using patch-clamp recording, glutamate sensor fluorescence imaging, immunohistochemistry, co-immunoprecipitation, Western blotting, and pharmacological methods.

## 2. Results

### 2.1. PGB Depressed Cerebellar PF-PC Synaptic Transmission via GluN2A-Containing NMDA Receptor

**Figure 1:**
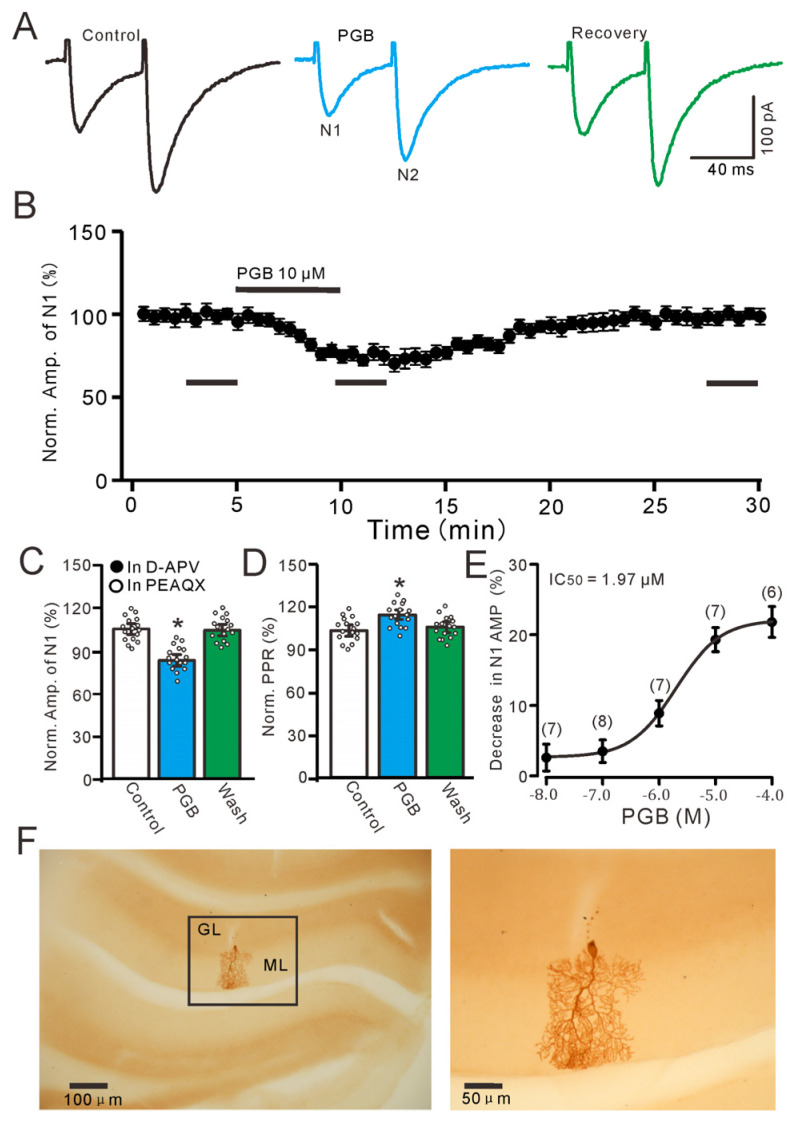
PGB depresses cerebellar PF–PC synaptic transmission in mouse brain slices. (**A**) Representative whole-cell patch-clamp traces of evoked EPSCs recorded from a Purkinje cell (PC) in response to paired-pulse stimulation (0.2 ms pulse duration, 50 ms inter-pulse interval) under control conditions, during pregabalin (PGB; 10 μM) application, and following washout (recovery). (**B**) Pooled data showing normalized N1 amplitude under these conditions. (**C**,**D**) Bar graphs with individual data points depicting normalized N1 amplitude (**C**) and paired-pulse ratio (PPR; (**D**)) for each treatment (black bars correspond to those in panel (**B**)). (**E**) Concentration–response curve for PGB-induced reduction in N1 amplitude, yielding an IC_50_ value of 1.97 μM. The number of recorded PCs for each concentration is indicated above the bars. (**F**) Low-magnification micrograph (left) and high-magnification inset (right) showing a biocytin-filled recorded PC. * *p* < 0.05 versus control; n = 16 cells/16 slices/10 mice.

To evaluate the effect of PGB on PF–PC synaptic transmission, excitatory postsynaptic currents (EPSCs) in PCs evoked by paired-pulse PF stimulation (0.2 ms, 10–100 µA; inter-pulse interval: 50 ms) were recorded in the presence of gabazine at a holding potential (Vh) of −70 mV. In the absence of GABA_A_ receptor activity, micro-application of PGB (10 µM) in the molecular layer induced a concentration-dependent depression of PF–PC EPSCs (Figure 1A,B). In the presence of PGB, the normalized N1 amplitude decreased to 88.2 ± 4.6% of the control level, which was significantly lower than under control conditions (100.0 ± 4.3%; F_(1,30)_ = 36.4, *p* < 0.0001; n = 16 cells/16 slices/10 mice; Figure 1C). The normalized PPR increased to 113.6 ± 2.5% of control, showing a significant elevation compared with the control group (100.0 ± 2.3%; F_(1,30)_ = 16.7, *p* = 0.0001; n = 16 cells/16 slices/10 mice; Figure 1D). To further determine whether PGB-induced suppression of PF–PC EPSCs exhibits sex-dependent differences, we compared the effects of PGB on N1 amplitude and PPR between male and female mice. In the presence of PGB, the normalized N1 amplitude in male mice was 88.4 ± 5.5% of control, a value comparable to that in female mice (88.0 ± 4.3%; F_(1,14)_ = 0.164, *p* = 0.76; n = 8 cells/8 slices/5 mice per group). The normalized PPR in male mice was 113.4 ± 2.9% of control, with no significant difference from the female counterpart (113.0 ± 2.7%; F_(1,14)_ = 0.231, *p* = 0.53; n = 8 cells/8 slices/5 mice per group). The PGB-induced reduction in N1 amplitude was concentration-dependent (Figure 1E), with an IC_50_ of 1.97 μM. Biocytin staining verified the morphological features of the recorded PCs (Figure 1F). These findings demonstrate that PGB induces a concentration-dependent reduction in PF–PC EPSC amplitude, accompanied by an increase in PPR in mouse cerebellar slices.

**Figure 2:**
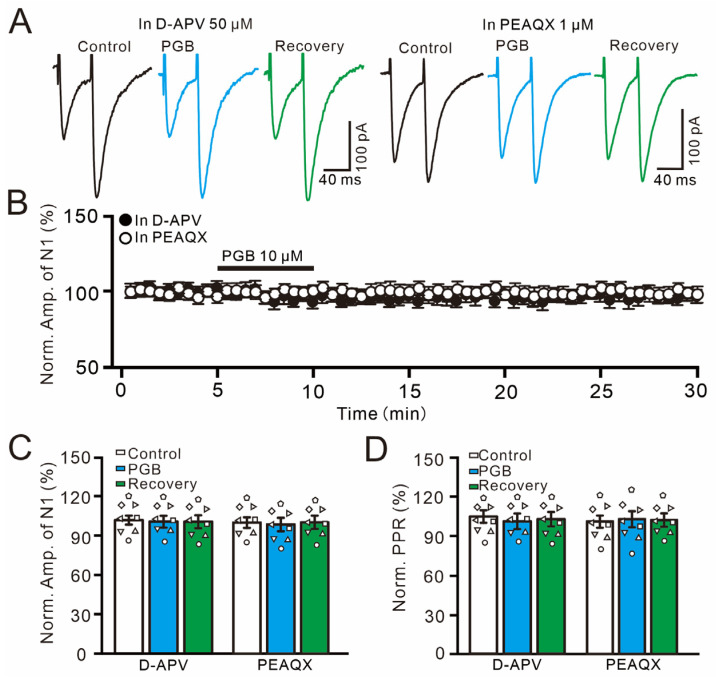
Blockade of either general NMDA receptors or GluN2A-containing NMDA receptors fully abolished the inhibitory effect of PGB on PF–PC synaptic transmission. (**A**) Representative traces of evoked EPSCs in response to paired-pulse stimulation (0.2 ms pulse duration, 50 ms inter-pulse interval) under control conditions, during pregabalin (PGB; 10 μM) application, and following washout (recovery), recorded in the presence of D-APV (left, 50 μM) or PEAQX (right, 1 μM). (**B**) Pooled data showing normalized N1 amplitude under these conditions. (**C**,**D**) Bar graphs with individual data points illustrating normalized N1 amplitude (**C**) and paired-pulse ratio (PPR; (**D**)) during ACSF, PGB perfusion, and washout. n = 8 cells/8 slices/6 mice for the D-APV group; n = 8 cells/8 slices/4 mice for the PEAQX group.

In the cerebellar cortex, parallel fibers (PFs) express functional NMDA receptors [37], which are critical for presynaptic plasticity at PF–PC synapses [29,38]. Notably, the α_2_δ-1 subunit selectively interacts with functional heteromeric NMDA receptors [16]. Gabapentinoids attenuate the hyperactivity of synaptic NMDA receptors by binding to the α_2_δ-1 subunit, thereby suppressing excessive excitatory synaptic transmission associated with neuropathic pain [39]. Therefore, we used the NMDA receptor antagonist D-APV to examine whether PGB-induced inhibition of PF–PC synaptic transmission is mediated by NMDA receptors. Following blockade of NMDA receptors by D-APV, PGB failed to depress PF–PC synaptic transmission (Figure 2A, left panel; Figure 2B). In the presence of D-APV (50 μM), the normalized N1 amplitude was 99.2 ± 4.8% of control, which was not significantly different from baseline (100.0 ± 3.1%; F_(1,14)_ = 0.173, *p* = 0.716; n = 8 cells/8 slices/6 mice; Figure 2C). The normalized PPR was 99.1 ± 5.3% of control, with no significant difference relative to baseline (100.0 ± 3.8%; F_(1,14)_ = 0.164, *p* = 0.743; n = 8 cells/8 slices/6 mice; Figure 2D).

In the cerebellar cortex, GluN2A-containing NMDA receptors are expressed on granule cell somata and PF terminals, where they modulate PF–PC synaptic transmission [40]. Therefore, we bath-applied the selective GluN2A-containing NMDA receptor antagonist PEAQX (1 μM) to determine whether PGB-induced inhibition of PF–PC synaptic transmission depends on GluN2A-containing NMDA receptors. Similar to D-APV, blockade of GluN2A-containing NMDA receptors with PEAQX abolished the inhibitory effect of PGB on PF–PC synaptic transmission (Figure 2A, right panel; Figure 2B). In the presence of PEAQX (1 μM), the normalized N1 amplitude was 98.1 ± 5.4% of control, with no significant difference from baseline values (100.0 ± 4.6%; F_(1,14)_ = 0.431, *p* = 0.521; n = 8 cells/8 slices/4 mice; Figure 2C). The normalized PPR was 101.3 ± 5.5% of control, which also showed no significant difference relative to control (100.0 ± 4.3%; F_(1,14)_ = 0.242, *p* = 0.629; n = 8 cells/8 slices/4 mice; Figure 2D). These findings demonstrate that PGB suppresses PF–PC synaptic transmission via inhibition of GluN2A-containing NMDA receptors in mouse cerebellar slices.

### 2.2. PGB Depressed Cerebellar PF-PC EPSCs via Presynaptic α*_2_*δ-1-NMDA Receptor Complexes

**Figure 3:**
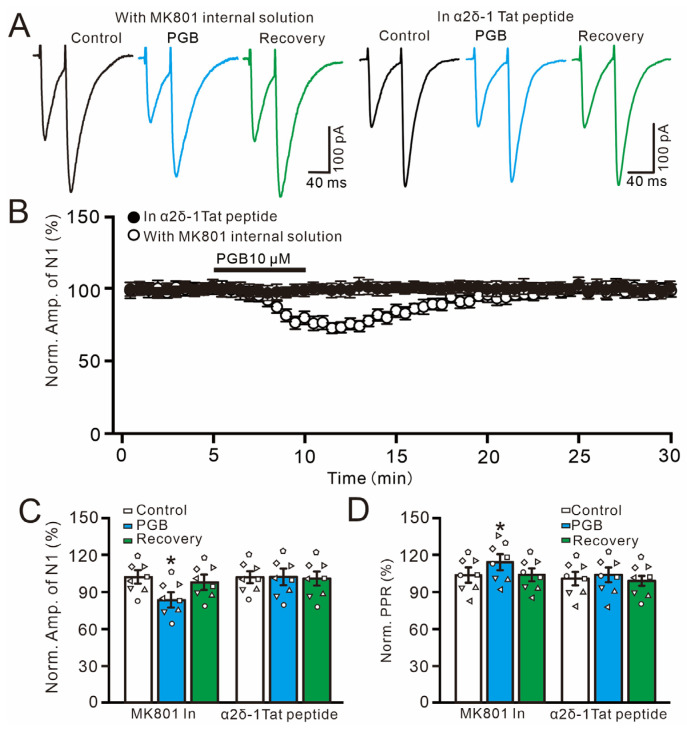
The inhibitory effect of PGB on PF–PC synaptic transmission persisted following blockade of postsynaptic NMDA receptors but was abolished by disruption of the α_2_δ-1–NMDAR interaction. (**A**) Representative traces of evoked EPSCs in response to paired-pulse stimulation (0.2 ms pulse duration, 50 ms inter-pulse interval) under control conditions, during pregabalin (PGB; 10 μM) application, and following washout (recovery). Recordings were performed with MK-801-containing pipette solution (left) or following extracellular application of the α2δ-1 Tat peptide (right; 1 μM). (**B**) Pooled data showing normalized N1 amplitude under these conditions. (**C**,**D**) Bar graphs with individual data points illustrating normalized N1 amplitude (**C**) and paired-pulse ratio (PPR; (**D**)) under these conditions. * *p* < 0.05 versus control; n = 8 cells/8 slices/5 mice for the MK-801 group; n = 8 cells/8 slices/6 mice for the α2δ-1 Tat peptide group.

To determine whether NMDA receptor inhibition occurs at presynaptic terminals or the postsynaptic membrane, we included the NMDA receptor blocker MK-801 (1 mM) in the pipette solution [41]. When PCs were recorded with pipette internal solution containing MK-801, micro-application of PGB in the molecular layer still depressed PF–PC synaptic transmission (Figure 3A,B). The normalized N1 amplitude under PGB treatment was 84.5 ± 6.8% of control, significantly lower than baseline (100.0 ± 6.5%; F(1,14) = 36.4, *p* < 0.0001; n = 8 cells/8 slices/5 mice; Figure 3C). Meanwhile, the normalized PPR reached 114.1 ± 6.2% of control, showing a significant increase relative to control (100.0 ± 6.1%; F(1,14) = 8.61, *p* = 0.026; n = 8 cells/8 slices/5 mice; Figure 3D). Collectively, these findings demonstrate that PGB-induced suppression of PF–PC synaptic transmission is independent of postsynaptic NMDA receptor activation, indicating that PGB exerts its inhibitory effect on PF–PC synaptic transmission through presynaptic NMDA receptors.

Given that α_2_δ-1 associates with NMDA receptors to form α_2_δ-1–NMDAR complexes [22,25], our present findings show that PGB-induced depression of PF–PC synaptic transmission is abolished by extracellular NMDA receptor blockade, implying that PGB suppresses PF–PC EPSCs via inhibition of α_2_δ-1–NMDAR complex activity. Therefore, we bath-applied an α_2_δ-1–NMDAR-disrupting peptide, the α_2_δ-1-Tat peptide (1 μM), to test whether disruption of α_2_δ-1–NMDAR complexes eliminates the inhibitory effect of PGB on PF–PC synaptic transmission. Notably, disruption of α_2_δ-1–NMDAR complexes completely abolished PGB-mediated suppression of PF–PC synaptic transmission (Figure 3A, right panel; Figure 3B). In the presence of the α_2_δ-1-Tat peptide (1 μM), normalized N1 amplitude was 100.2 ± 6.3% of control, with no significant difference relative to baseline (100.0 ± 5.1%; F_(1,14)_ = 0.243, *p* = 0.657; n = 8 cells/8 slices/6 mice; Figure 3C). Normalized PPR was 101.9 ± 6.1% of control, also showing no significant difference from baseline (100.0 ± 4.8%; F_(1,14)_ = 0.351, *p* = 0.547; n = 8 cells/8 slices/6 mice; Figure 3D). Collectively, these results demonstrate that PGB depresses PF–PC synaptic transmission by inhibiting presynaptic α_2_δ-1–NMDAR complexes in mouse cerebellar slices.

### 2.3. PGB Depresses mEPSCs via Presynaptic α*_2_*δ-1-NMDA Receptor Complexes

**Figure 4:**
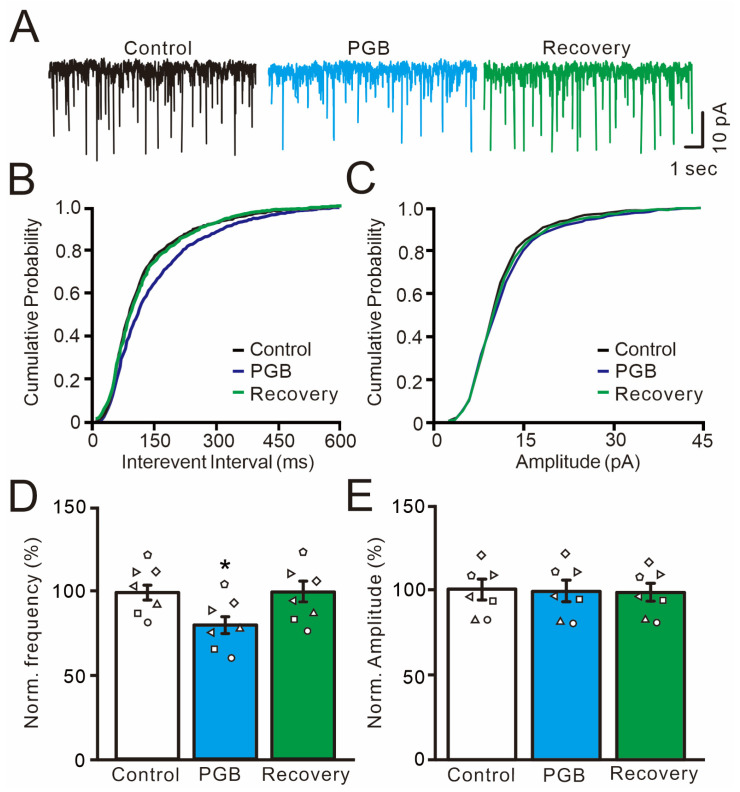
Effect of PGB on mEPSCs in cerebellar PCs. (**A**) Representative membrane current traces recorded from a cerebellar PC in the presence of gabazine (20 μM) and TTX (1 μM) under control conditions, during pregabalin (PGB; 10 μM) application, and following washout (recovery). (**B**) Cumulative probability curves of mEPSC interevent intervals under control, PGB-treated, and washout (recovery). (**C**) Cumulative probability curves of mEPSC amplitudes under control, PGB-treated, and washout (recovery) conditions. (**D**,**E**) Bar graphs displaying mean ± S.E.M. with individual data points for normalized mEPSC frequency (**D**) and amplitude (**E**) in PCs under control, PGB-treated, and washout (recovery) conditions. * *p* < 0.05 versus control; n = 7 cells/7 slices/5 mice.

To determine whether PGB suppresses glutamatergic synaptic transmission at parallel-fiber terminals, we examined its effects on miniature excitatory postsynaptic currents (mEPSCs) in mouse cerebellar slices. To record mEPSCs from PCs, gabazine (20 μM) and tetrodotoxin (TTX; 1 μM) were added to ACSF to block spontaneous action potentials and GABAergic inhibitory inputs [31]. Under voltage-clamp recordings at a holding potential of −70 mV, micro-application of PGB in the molecular layer significantly reduced mEPSC frequency (Figure 4A), accompanied by a leftward shift in the cumulative probability curve of interevent intervals (Figure 4B). In contrast, PGB exerted no significant effect on mEPSC amplitude; the cumulative amplitude probability curve remained unchanged, with no significant difference in mEPSC amplitude before and after PGB application (Figure 4C). In the presence of PGB, normalized mEPSC frequency decreased to 81.6 ± 6.1% of control (100.0 ± 5.6%; F_(1,12)_ = 23.42, *p* < 0.0001; n = 7 cells/7 slices/5 mice; Figure 4D), whereas normalized mEPSC amplitude remained at 99.5 ± 7.6% of control (100.0 ± 4.8%; F_(1,12)_ = 0.186, *p* = 0.674; n = 7 cells/7 slices/5 mice; Figure 4E). These findings demonstrate that PGB reduces mEPSC frequency but not amplitude, consistent with a presynaptic mechanism whereby PGB inhibits glutamate release from PF terminals.

**Figure 5:**
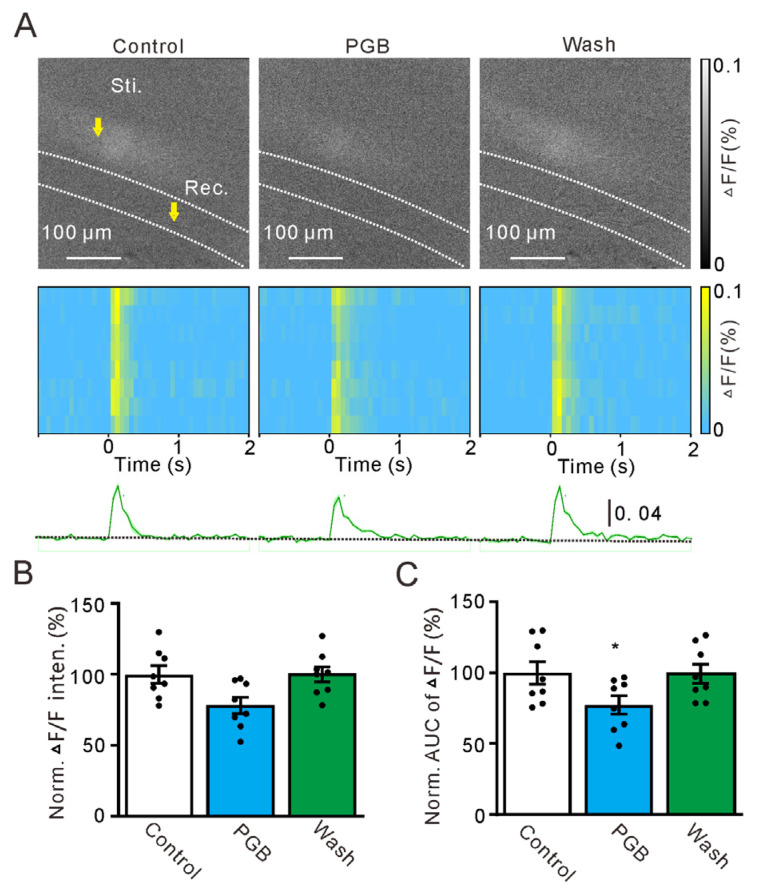
Effects of PGB on electrically evoked PF–PC-associated glutamate fluorescence within the cerebellar molecular layer. (**A**) Upper panel: Representative images displaying iGluSnFR fluorescence signals upon PF stimulation. Yellow arrows mark the positions of the stimulating electrode (Sti., placed in the molecular layer, ML) and the recording site (Rec., within the ML). Middle panel: Individual heatmaps of PF stimulation-evoked (50 ms, 2 pulses) iGluSnFR fluorescence responses in the ML under control, PGB-treated, and washout conditions. Lower panel: Averaged iGluSnFR fluorescence traces from eight trials, with baseline drift corrected. (**B**,**C**) Bar graphs with individual data points summarizing normalized ΔF/F intensity (**B**) and area under the curve (AUC; C) of iGluSnFR signals across control, PGB-treated, and washout groups. * *p* < 0.05 vs. Control. n = 8 recordings from 4 mice per group. Sti., stimulating electrode; Rec., recording site.

To clarify whether PGB-induced suppression of PF–PC synaptic transmission arises from reduced glutamate release at PF terminals, we used the optimized glutamate sensor iGluSnFR.A184S to monitor glutamatergic signaling dynamics in acute mouse cerebellar slices [42]. As shown in Figure 5, paired-pulse stimulation of PFs reliably evoked band-like transient increases in iGluSnFR fluorescence within the molecular layer, and this signal was significantly attenuated following bath application of PGB (10 μM; Figure 5A). In the presence of PGB, the normalized intensity of PF-evoked iGluSnFR signals decreased to 78.3 ± 5.8% of control (100.0 ± 6.2%; F(1,7) = 11.0, *p* = 0.01; n = 8 recordings/4 mice; Figure 5A,B). Meanwhile, the normalized area under the curve (AUC) of evoked fluorescence responses was reduced to 77.4 ± 6.5% of control (100.0 ± 8.0%; F(1,7) = 6.5, *p* = 0.04; n = 8 recordings/4 mice; Figure 5A,C). Collectively, these findings demonstrate that PGB dampens PF-evoked glutamate-dependent iGluSnFR signals at PF–PC synapses, indicating that PGB depresses PF–PC synaptic transmission by inhibiting presynaptic glutamate release in the mouse cerebellar cortex.

**Figure 6:**
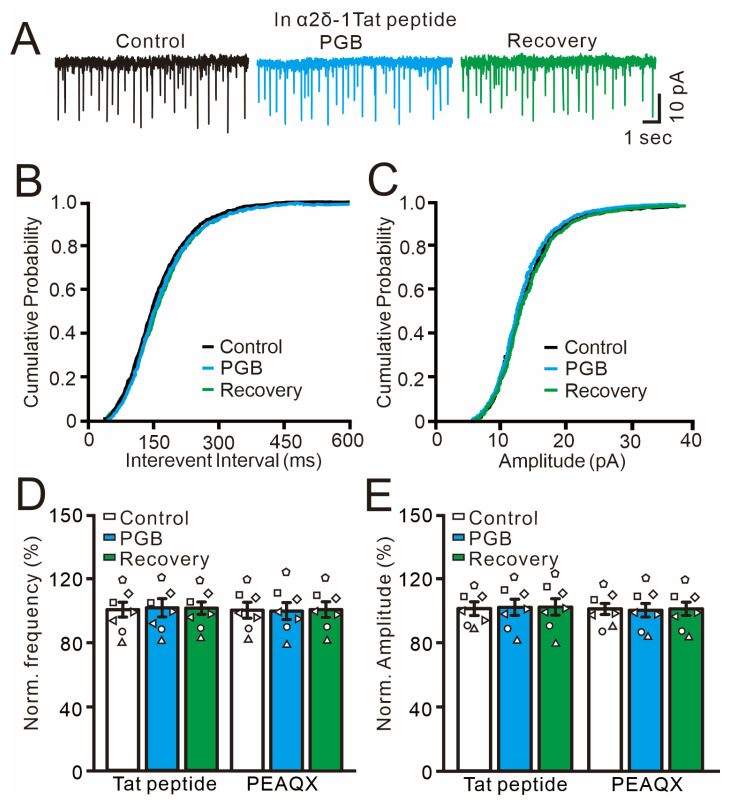
The effect of PGB on mEPSCs was prevented by blockade of GluN2A-containing NMDA receptors or disruption of α_2_δ-1–NMDA receptor coupling. (**A**) In the presence of a mixture of gabazine (20 μM), TTX (1 μM), and α2δ-1Tat peptide (1 μM), representative membrane current traces of a cerebellar PC recorded under control conditions, during pregabalin (PGB; 10 μM) application, and following washout (recovery). (**B**,**C**) In the presence of α2δ-1Tat peptide, cumulative probability–interevent interval curves (**B**) and cumulative probability–amplitude curves (**C**) of mEPSCs under control, PGB treatment, and recovery conditions. (**D**,**E**) Bar graphs showing the mean ± S.E.M. and individual data points for the normalized mEPSC frequency (**D**) and amplitude (**E**) in PCs in the presence of α2δ-1Tat peptide (1 μM; left panel) and PEAQX (1 µM; right panel) under each condition. n = 7 cells/7 slices/4 mice in PEAQX group; n = 7 cells/7 slices/6 mice in α2δ-1Tat peptide group.

Since PGB-mediated suppression of PF–PC EPSCs was abolished by either disruption of α_2_δ-1–NMDAR complexes or blockade of GluN2A-containing NMDA receptors, we further investigated whether PGB regulates mEPSCs by modulating α_2_δ-1-GluN2A-containing NMDA receptor complexes. Either disruption of α_2_δ-1–NMDAR complexes or antagonism of GluN2A-containing NMDA receptors completely abolished the inhibitory effect of PGB on mEPSCs in Purkinje cells (PCs) (Figure 6). In the presence of the α_2_δ-1-Tat peptide (1 μM), PGB failed to reduce mEPSC frequency (Figure 6A), with no significant shift observed in the cumulative probability curves for mEPSC frequency (Figure 6B) and amplitude (Figure 6C). When co-applied with the α_2_δ-1-Tat peptide, PGB did not alter normalized mEPSC frequency (101.2 ± 6.1% of control; 100.0 ± 4.9%; F(1,12) = 0.263, *p* = 0.462; n = 7 cells/7 slices/4 mice; Figure 6D) or amplitude (100.3 ± 5.9% of control; 100.0 ± 4.6%; F(1,12) = 0.135, *p* = 0.761; n = 7 cells/7 slices/4 mice; Figure 6E). Similarly, co-application of PEAQX with PGB had no significant effect on normalized mEPSC frequency (99.8 ± 5.9% of control; 100.0 ± 5.6%; F(1,12) = 0.137, *p* = 0.764; n = 7 cells/7 slices/6 mice; Figure 6D) or amplitude (99.7 ± 5.7% of control; 100.0 ± 5.2%; F(1,12) = 0.118, *p* = 0.773; n = 7 cells/7 slices/6 mice; Figure 6E). These findings demonstrate that PGB-induced reduction in mEPSC frequency is abolished by either disruption of α_2_δ-1–NMDAR complexes or antagonism of GluN2A-containing NMDA receptors, indicating that PGB modulates mEPSCs via α_2_δ-1-GluN2A-containing NMDA receptor complexes.

### 2.4. Presynaptic PKA Activation Mediates PGB-Induced Suppression of PF–PC Synaptic Transmission

**Figure 7:**
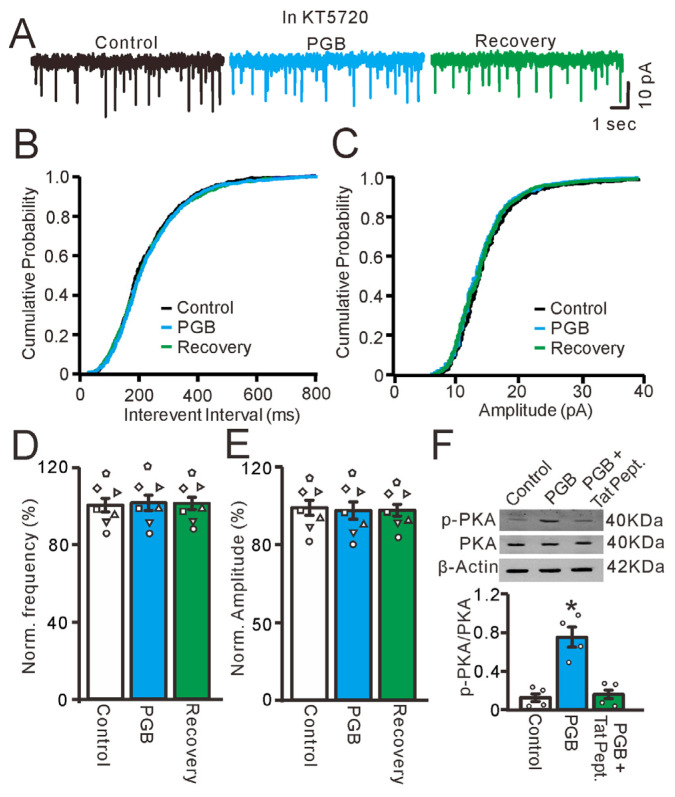
PKA inhibition completely prevented the effect of PGB on mEPSCs. (**A**) In the presence of a mixture of gabazine (20 μM), TTX (1 μM), and KT5720 (100 nM), representative membrane current traces of a cerebellar PC recorded under control conditions, during pregabalin (PGB; 10 μM) application, and following washout (recovery). (**B**) Cumulative probability–interevent interval curves of mEPSCs under control, PGB treatment, and recovery conditions. (**C**) Cumulative probability–amplitude curves of mEPSCs under control, PGB treatment, and recovery conditions. (**D**,**E**) Bar graphs showing the mean ± S.E.M. and individual data points for the normalized mEPSC frequency (**D**) and amplitude (**E**) in PCs under control, PGB treatment, and recovery conditions. n = 7 cells/7 slices/6 mice. (**F**) Upper, representative Western blot bands show the protein levels of PKA, p-PKA and β-Actin in the mouse cerebellar cortical molecular layer tissues among groups of control, PGB and α2δ-1Tat peptide + PGB; lower, the densitometric quantification of p-PKA/PKA in each group. * *p* < 0.05 versus control; n = 4 mice in each group.

We further investigated whether PGB decreases mEPSC frequency via activation of the PKA signaling pathway. In the presence of KT5720 (100 nM), micro-application of PGB (10 µM) failed to reduce mEPSC frequency and had no significant effect on the cumulative probability curves for either mEPSC frequency (Figure 7A,B) or amplitude (Figure 7C). In the presence of the KT5720 and PGB, the normalized frequency of mEPSCs was 100.3 ± 4.7% of the control condition (100.0 ± 3.8%; F _(1,12)_ = 0.106, *p* = 0.719; n = 7 cells/7 slices/6 mice; Figure 7D), and the normalized amplitude of mEPSCs was 99.6 ± 4.9% of the control condition (100.0 ± 4.8%; F _(1,12)_ = 0.135, *p* = 0.635; n = 7 cells/7 slices/6 mice; Figure 7E). These findings demonstrate that PGB significantly inhibits presynaptic glutamate release through the PKA signaling pathway, thereby reducing mEPSC frequency in PCs.

Western blot analysis was performed to assess the protein levels of PKA, p-PKA, and β-actin in the molecular layer of the mouse cerebellar cortex across the control, PGB, and α2δ-1 Tat peptide + PGB groups. The results showed that PGB treatment markedly increased p-PKA expression (Figure 7F). Densitometric quantification of the p-PKA/PKA ratio yielded a value of 0.72 ± 0.21 in the PGB group, which was significantly higher than that in the control group (0.11 ± 0.023; F (1,6) = 12.92, *p* = 0.011; n = 4). By contrast, the p-PKA/PKA ratio in the α2δ-1 Tat peptide + PGB group was 0.15 ± 0.025 (F_(1,6)_ = 0.142, *p* = 0.68; n = 4), with no obvious difference compared with the control group. These findings indicate that PGB enhances PKA phosphorylation via activation of α_2_δ-1-NMDAR complexes, thereby upregulating p-PKA levels.

**Figure 8:**
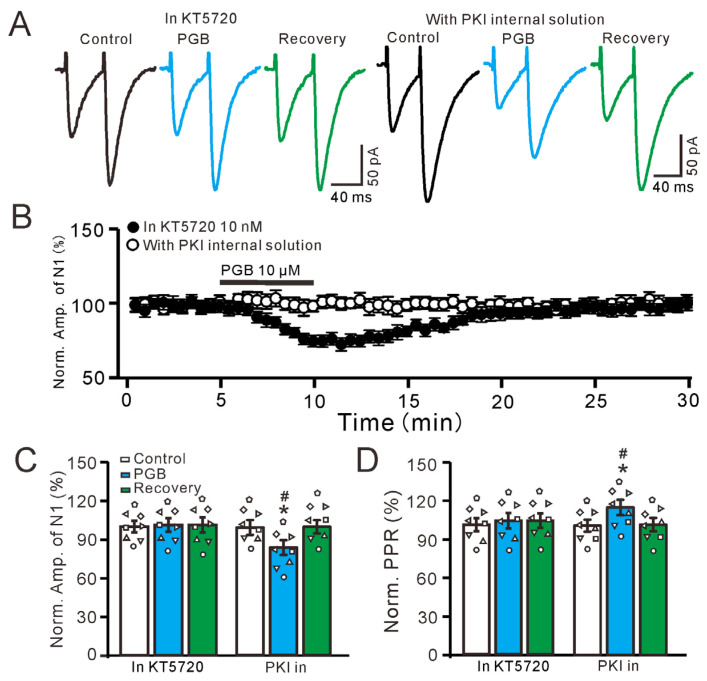
PGB-induced suppression of PF–PC synaptic transmission was abolished by extracellular, but not intracellular, PKA inhibition. (**A**) In the presence of PKA inhibitor, KT5720 (left; 100 nM) and recording PCs with PKI internal solution (right), representative traces show evoked EPSCs in response to paired-pulse stimulation (duration: 0.2 ms, interval: 50 ms) under control conditions, during pregabalin (PGB; 10 μM) application, and following washout (recovery). (**B**) Pooled data showing the normalized amplitude of N1 during these treatments. (**C**,**D**) Bar graphs with individual data points show the normalized amplitude of N1 (**C**) and the paired-pulse ratio (PPR; (**D**)) during perfusion with ACSF, pregabalin (PGB), and the recovery period. n = 8 cells/8 slices/5 mice in KT5720 group; n = 8 cells/8 slices/5 mice in PKI group. * *p* < 0.05 versus control; # *p* < 0.05 versus PGB in KT5720 group.

Moreover, we used the selective PKA inhibitor KT5720 (100 nM) to investigate whether PGB-induced suppression of PF–PC synaptic transmission is mediated by PKA activation. To ensure full PKA inhibition, KT5720 was bath-applied for 30 min prior to whole-cell patch-clamp recordings from PCs. With the inhibition of PKA activity, micro-application of PGB in the molecular layer failed to decrease the PF-PC EPSCs (Figure 8A,B). In the presence of KT5720 and PGB, the normalized amplitude of N1 was 100.3 ± 6.6% of the control condition (100.0 ± 4.5%; F_(1,14)_ = 0.106, *p* = 0.781; n = 8 cells/8 slices/5 mice; Figure 8C), and the normalized PPR was 102.3 ± 6.9% of the control condition (100.0 ± 5.6%; F_(1,14)_ = 0.531, *p* = 0.437; n = 8 cells/8 slices/5 mice; Figure 8D). These results indicate that inhibition of PKA abolished the PGB-induced depression of PF-PC synaptic transmission.

To determine whether PKA inhibition acts at presynaptic terminals or postsynaptic sites, we included protein kinase inhibitor-(6-22) amide (PKI) in the patch pipette solution to inhibit postsynaptic PKA in PCs [43]. Inhibition of intracellular PKA activity in PCs did not alter the PGB-induced depression of PF–PC EPSCs (Figure 8A,B). In the presence of PGB, the normalized N1 amplitude was 83.1 ± 7.2% of the control value, which was significantly lower than that under control conditions (100 ± 7.1%; F_(1,14)_ = 12.1, *p* = 0.003; n = 8 cells/8 slices/5 mice; Figure 8C). In addition, the normalized PPR was 113.7 ± 6.8% of the control value, which showed no significant difference compared with that under control conditions (100.0 ± 5.7%; F_(1,14)_ = 9.35, *p* = 0.021; n = 8 cells/8 slices/5 mice; Figure 8D). These results indicate that PGB impairs PF–PC synaptic transmission via a presynaptic PKA signaling pathway, suggesting that PGB reduces glutamate release from PF terminals.

**Figure 9:**
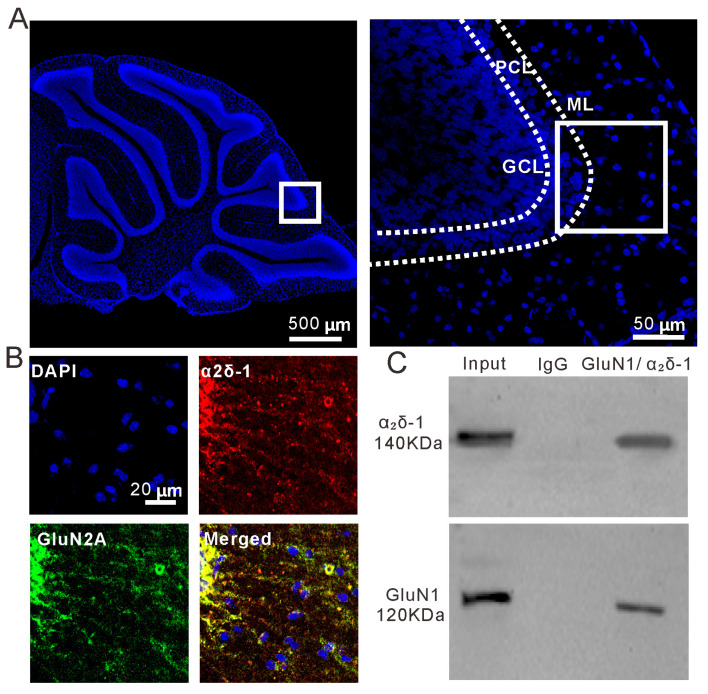
Co-expression of α_2_δ-1 and GluN2A subunits in the molecular layer of the mouse cerebellum. (**A**) Left panel: confocal micrograph showing DAPI staining (blue) in the mouse cerebellar cortex; right panel: magnified view of DAPI staining (blue) corresponding to the boxed area in the left panel. DAPI is a blue nucleic acid dye that preferentially stains double-stranded DNA (dsDNA) in cells. (**B**) Higher-magnification images of the boxed area in the right panel of (**A**), showing DAPI staining (blue), α_2_δ-1 subunit immunoreactivity (red), GluN2A immunoreactivity (green), and the merged immunofluorescence image in the Purkinje cell layer (PCL) and molecular layer (ML). Red fluorescence signals for the α_2_δ-1 subunit and green fluorescence signals for GluN2A immunoreactivity were detected in the ML, surrounding the dendrites of PCs. ML, molecular layer; PCL, Purkinje cell layer; GCL, granular cell layer. (**C**) Co-immunoprecipitation analysis revealed the protein–protein interaction between α_2_δ-1 and NMDAR in membrane extracts from the molecular layer of the cerebellar cortex. Proteins were first immunoprecipitated with rabbit anti-GluN1, anti-α_2_δ-1, or control IgG antibodies. Western immunoblotting (IB) was then performed using mouse anti-α_2_δ-1 (upper panel) and anti-GluN2A antibodies (lower panel). IgG and input samples (tissue lysates without immunoprecipitation) served as the negative and positive controls, respectively.

Finally, we examined the expression of the α_2_δ-1 subunit and GluN2A-containing NMDA receptors in the mouse cerebellar cortex using confocal laser-scanning microscopy (A1RMP, Nikon, Tokyo, Japan). As shown in Figure 9, immunoreactivity for both α_2_δ-1 and GluN2A was detected in the molecular layer, with robust expression around the dendrites of PCs (Figure 9A,B). We further performed co-immunoprecipitation to examine the protein–protein interaction between α_2_δ-1 and NMDAR in membrane extracts from the molecular layer of the cerebellar cortex. Proteins were first immunoprecipitated using rabbit anti-GluN1, anti-α_2_δ-1, or control IgG antibodies. Subsequent Western immunoblotting was performed with mouse anti-α_2_δ-1 or anti-GluN2A antibodies. The results showed that α_2_δ-1 and GluN1 were mutually pulled down in tissue samples from the mouse cerebellar molecular layer, indicating that the α_2_δ-1-NMDA receptor complex exists in the molecular layer of the mouse cerebellar cortex (Figure 9C). These findings provide molecular evidence that α_2_δ-1 subunits couple with GluN2A-containing NMDA receptors within the cerebellar cortical molecular layer of mice.

## 3. Discussion

The present study demonstrates that PGB reduces the amplitude of cerebellar PF–PC EPSCs, accompanied by an increased PPR. PGB-induced suppression of PF–PC EPSCs was abolished by extracellular blockade of GluN2A-containing NMDA receptors or disruption of the α_2_δ-1–NMDAR interaction, but not by postsynaptic intracellular NMDA receptor inhibition. PGB decreased mEPSC frequency and glutamate sensor fluorescence intensity; these effects were abrogated by blockade of GluN2A-containing NMDA receptors and disruption of α_2_δ-1–NMDAR coupling. Furthermore, the inhibitory actions of PGB on both PF–PC EPSCs and mEPSCs were eliminated by presynaptic—not postsynaptic—PKA inhibition. Western blot analysis revealed that PGB elevated PKA phosphorylation in the molecular layer of the mouse cerebellar cortex. Immunohistochemistry demonstrated abundant co-expression of GluN2A and α_2_δ-1 subunits around PC dendrites within the mouse cerebellar cortex. Co-immunoprecipitation further confirmed that α_2_δ-1 was co-precipitated with GluN1 in tissue samples from the mouse cerebellar molecular layer. Collectively, these findings demonstrate that PGB suppresses glutamate release from parallel-fiber terminals via the presynaptic α_2_δ-1–GluN2A-containing NMDA receptor-dependent PKA signaling pathway in the mouse cerebellar cortex.

### 3.1. PGB Suppresses PF–PC Synaptic Transmission by Modulating Presynaptic GluN2A-Containing NMDA Receptors

PGB is a structural derivative of GABA and is widely used clinically for the management of peripheral neuropathic pain and as an adjunctive treatment for epileptic seizures [1,2,3]. PGB expresses binding affinity for the α_2_δ subunits of VDCCs, with preferential binding to the α_2_δ-1 isoform over α_2_δ-2 [5,8,9]. Previous studies have shown that PGB interacts with α_2_δ subunits of VGCCs in the cerebral cortex, hippocampus, and cerebellum, indicating that PGB modulates neuronal excitability and synaptic transmission across broad brain regions [5,13,14]. By binding to α_2_δ auxiliary subunits of VGCCs, PGB regulates multiple forms of synaptic transmission via modulating the presynaptic release of neurotransmitters including glutamate and GABA [4,5,6,7]. Functional NMDA receptors are expressed at cerebellar parallel-fiber (PF) terminals, where they critically contribute to PF-PC synaptic transmission and long-term synaptic plasticity [29,37,38]. Importantly, the α_2_δ-1 subunit potentiates NMDA receptor activity and facilitates NMDA receptor-mediated glutamatergic synaptic transmission [21], and selectively interacts with functional heteromeric NMDARs in rat dorsal root ganglion neurons [22]. PGB binds to the α_2_δ-1 subunit, suppressing NMDA receptor activation [44] and normalizing hyperactive synaptic NMDARs by inhibiting α_2_δ-1 channel activity, which in turn attenuates excessive excitatory synaptic transmission underlying neuropathic pain [40]. In the present study, micro-application of PGB to the molecular layer elicited a concentration-dependent decrease in PF-PC EPSC amplitude, accompanied by an increased paired-pulse ratio (PPR), indicating that PGB depresses PF-PC synaptic transmission through a presynaptic mechanism. Furthermore, when postsynaptic NMDA receptor activity in Purkinje cells (PCs) was inhibited via intracellular dialysis with MK-801, PGB failed to depress PF–PC synaptic transmission, demonstrating that PGB-induced synaptic suppression is independent of postsynaptic NMDA receptor activation. In addition, PGB markedly reduced mEPSC frequency and glutamate sensor fluorescence intensity, consistent with reduced glutamate release from PF terminals [42,43]. Collectively, these findings support that PGB targets presynaptic NMDA receptors to reduce glutamate release, thereby suppressing PF–PC synaptic transmission in mouse cerebellar slices.

In the cerebellar cortex, GluN2A-containing NMDA receptors are expressed on granule cell somata and their axonal terminals, where they modulate PF–PC synaptic transmission and long-term synaptic plasticity [37]. Confocal laser-scanning microscopy further demonstrated strong immunofluorescent signals for GluN2A-containing NMDA receptors in both the granular and molecular layers of the mouse cerebellar [40,45]. The present data confirm our previous findings [40,45], demonstrating abundant expression of GluN2A-containing NMDA receptors in the mouse cerebellar cortex—particularly around the somata and dendrites of Purkinje cells (PCs). Furthermore, application of a selective GluN2A antagonist abolished PGB-induced suppression of PF–PC synaptic transmission, indicating that PGB depresses PF–PC synaptic transmission by inhibiting GluN2A-containing NMDA receptors. Moreover, the PGB-induced reduction in mEPSC frequency was also prevented by blockade of GluN2A-containing NMDA receptors, suggesting that PGB’s effect on mEPSCs is dependent on presynaptic GluN2A-containing NMDA receptors. Collectively, these results demonstrate that micro-application of PGB in the molecular layer inhibits PF–PC synaptic transmission in vitro in mouse cerebellar slices by reducing presynaptic glutamate release via modulation of presynaptic GluN2A-containing NMDA receptor activity.

### 3.2. PGB Depresses PF–PC Synaptic Transmission via Presynaptic α*_2_*δ-1-NMDA Receptor Complexes

The α_2_δ-1 subunit of VGCCs binds to NMDA receptors to form heteromeric α_2_δ-1–NMDAR complexes in the cell membrane; these complexes are critical for neuropathic pain pathogenesis and the therapeutic effects of gabapentinoids [22,39]. The α_2_δ-1 subunit interacts with NMDA receptors through its C-terminal domain to enhance NMDA receptor functional activity [44], and modulation of the α_2_δ-1 subunit regulates NMDA receptor channel function [24,25]. Binding of PGB to the α_2_δ-1–NMDAR complex inhibits NMDA receptor activity [46], reduces the NMDA-to-non-NMDA current ratio, and shortens the decay time of NMDA receptor-mediated EPSCs [47]. Our present findings demonstrate that PGB suppresses PF–PC synaptic transmission by targeting presynaptic NMDA receptors, implying that PGB reduces PF–PC EPSC amplitude through inhibition of α_2_δ-1–NMDAR complex function. In vitro electrophysiological experiments further revealed that disruption of α_2_δ-1–NMDAR complexes using the α_2_δ-1-Tat peptide [22,25] fully abolished PGB-induced suppression of PF–PC synaptic transmission in acute mouse cerebellar slices. Notably, the PGB-evoked reduction in mEPSC frequency was completely blocked by α_2_δ-1–NMDAR complex disruption, indicating that PGB attenuates presynaptic glutamate release via modulation of α_2_δ-1-GluN2A-containing NMDA receptor complexes.

### 3.3. PGB Suppresses PF–PC Synaptic Transmission Through the Presynaptic PKA Signaling Cascade

Previous studies have shown that gabapentin, an analog of PGB, enhances renal outer medullary potassium channel activity via PKA-mediated phosphorylation-driven conformational changes rather than charge–charge interactions [48]. Moreover, PGB-evoked potentiation of renal outer medullary potassium channel activity relies on either PKA- or PKC-dependent phosphorylation [49]. The PKA signaling pathway is well known to critically regulate neurotransmitter synthesis and release; accordingly, PGB may modulate presynaptic glutamate release and neurotransmitter vesicle exocytosis through the cAMP-PKA signaling cascade. We therefore further investigated whether PGB-induced suppression of PF–PC synaptic transmission depends on PKA activity. Our results revealed that following PKA inhibition, micro-application of PGB in the molecular layer no longer reduced PF-PC EPSC amplitude, demonstrating that PGB-induced suppression of PF-PC synaptic transmission relies on the presynaptic PKA signaling cascade. To determine whether PKA inhibition acts at the presynaptic terminal or postsynaptic site, we next incorporated the protein kinase inhibitor-(6-22) amide (PKI) into the patch pipette solution to inhibit postsynaptic PKA in PCs [43]. Inhibition of intracellular PKA activity in PCs did not alter PGB-induced suppression of PF–PC EPSCs, indicating that PGB impairs PF–PC synaptic transmission via the presynaptic PKA signaling pathway [43]. Moreover, PGB failed to reduce mEPSC frequency following PKA inhibition, demonstrating that PGB significantly decreases glutamate release through the PKA signaling pathway, thereby leading to a marked reduction in mEPSC frequency in PCs. Consistent with previous studies [48,49], the present findings demonstrate that micro-application of PGB to the cerebellar molecular layer significantly reduces glutamate release via the α_2_δ-1 subunit-coupled GluN2A-containing NMDA receptor/PKA signaling pathway, resulting in marked suppression of PF–PC synaptic transmission in in vitro mouse cerebellar slices. Our results suggest that PGB binds to α_2_δ-1 subunit-coupled GluN2A-containing NMDA receptors and downregulates adenylate cyclase activity at PF terminals, leading to decreased intracellular cAMP levels and inhibited PKA activity; this, in turn, attenuates PF–PC synaptic transmission. These findings provide novel insights into the mechanisms underlying PGB-mediated modulation of central nervous system function, and suggest that PGB modulates cerebellar PC output, thereby influencing motor coordination and motor learning in vivo.

### 3.4. Clinical Significance

Cerebellar PCs primarily receive two excitatory inputs from PFs and climbing fibers, and serve as the sole output to the deep cerebellar nuclei, supporting diverse functional processes including motor planning, execution, and coordination [28]. PFs are axons of granule cells that receive information from mossy fibers and form excitatory synapses with PCs and molecular layer interneurons (MLIs). Information transmitted through the mossy fiber–granule cell–PF pathway is primarily relayed by glutamate released from PF terminals, which induces excitatory synaptic transmission and plasticity at PF–PC synapses [29]. Notably, PF–PC synaptic transmission and plasticity play critical roles in regulating fine motor control and motor learning [28,29]. The present findings demonstrate that PGB reduces glutamate release via α_2_δ-1 subunit-coupled GluN2A-containing NMDA receptors, leading to marked suppression of PF–PC synaptic transmission in the mouse cerebellar cortex. Of note, PGB exhibits particularly high affinity for α_2_δ-2 subunit-containing VGCCs in the cerebellum, suggesting that PGB may exert diverse modulatory effects on cerebellar circuits [35]. Collectively, our results indicate that PGB modulates cerebellar cortical circuit function by suppressing PF–PC synaptic transmission, implying that PGB administration may impair cerebellar functions such as fine motor control and motor coordination [28,29]. Furthermore, clinical evidence has shown that PGB treatment can induce various movement disorders in humans, including ataxia, tremors, myoclonus, and parkinsonism [36]. PGB depresses PF–PC synaptic transmission via α_2_δ-1 subunit-coupled GluN2A-containing NMDA receptors; this mechanism may underlie PGB-related cerebellar dysfunction, thereby accounting for the ataxia, tremors, and myoclonus observed in patients receiving PGB treatment.

### 3.5. Limitations and Future Directions

The cerebellar cortical circuit is primarily composed of PCs, MLIs, granule cells, and Golgi cells. Cerebellar PCs receive two main excitatory inputs—PFs and climbing fibers—and serve as the sole output of the cerebellar cortex to the deep cerebellar nuclei, regulating multiple physiological functions including motor planning, execution, and coordination [28]. In the intact cerebellum, sensory information transmitted through the mossy fiber–granule cell–PF pathway excites MLIs, which in turn suppress PC activity. Sensory input can only evoke PC excitation when GABAA receptor activity is blocked, indicating that the MLI network plays a critical role in modulating PC excitability and PF–PC synaptic transmission in the intact cerebellar cortex [50,51]. Since the electrophysiological recordings in the present study were performed in acute cerebellar slices, tissue slicing inevitably disrupts the intrinsic MLI network. Under in vitro conditions, PF–PC synaptic transmission was evoked by direct electrical stimulation of PFs, with GABAergic transmission absent. Accordingly, the PGB-induced suppression of PF–PC synaptic transmission observed in cerebellar slices differs from the sensory stimulation-evoked synaptic transmission in intact animals. Therefore, to fully elucidate the effects of PGB on cerebellar circuit function, future studies are needed to investigate PGB-mediated modulation of the activity of PCs, MLIs, and granule cells, as well as synaptic transmission along the sensory stimulation-evoked mossy fiber–granule cell–PF pathway in living animals. Additionally, the impacts of PGB application within the cerebellar cortex on fine motor control and motor learning should be evaluated in subsequent behavioral experiments, such as the rotarod test.

## 4. Materials and Methods

### 4.1. Animals

A total of 94 C57BL/6J mice (6–8 weeks old; equal numbers of males and females) were obtained from the Animal Center of Yanbian University. Mice were group-housed in a stable housing environment with a constant temperature of 24 ± 1 °C and a relative humidity of 50 ± 5%. A 12-h light/dark cycle was adopted (light on: 6:00–18:00), and the mice had ad libitum access to food and water. All experimental procedures were approved by the Animal Care and Use Committee of Yanbian University, with the following license number: SYXK (Ji) 2025-006 (approval date: 11 July 2025). All experimental methods complied with the Animal Welfare Guidelines for Laboratory Animals of the National Institutes of Health (NIH) and followed Animal Research: Reporting of In Vivo Experiments (ARRIVE; https://arriveguidelines.org (accessed on 14 July 2020)).

### 4.2. Experimental Design

The mice were randomly assigned to the control group and the PGB treatment group. Whole-cell patch-clamp recording combined with pharmacological methods was performed on acute cerebellar slices to explore the regulatory mechanism of PGB on PF–PC synaptic transmission. Immunohistochemistry and confocal fluorescence imaging, together with co-immunoprecipitation, were applied to detect the co-localization and expression of GluN2A and α_2_δ-1 subunits in the cerebellar cortex. Western blot analysis was performed to examine the expression levels of PKA and p-PKA in cerebellar tissues before and after PGB application. Finally, glutamate sensor fluorescence imaging was used to assess the effect of PGB on glutamate release from PFs onto PCs.

### 4.3. Cerebellar Slice Preparation

Seventy-four mice were deeply anesthetized with halothane and quickly decapitated for the preparation of cerebellar slices. The dissected whole brain was placed in ice-cold artificial cerebrospinal fluid (ACSF) containing the following components (in mM): 125 sodium chloride, 3 potassium chloride, 1 magnesium sulfate, 2.5 calcium chloride, 1 sodium dihydrogen phosphate, 25 sodium bicarbonate, and 10 glucose. It was given a continuous flow of 95% oxygen/5% carbon dioxide mixed gas (pH 7.4; osmotic pressure 295–305 mOsm). After the brain was completely cooled, 250 µm thick sagittal slices of the cerebellar cortex were prepared using a Leica Vibratome (Leica VT 1200s; Leica Biosystems Nussloch GmbH, Nussloch, Germany). The cerebellar slices were incubated in a chamber containing 95% oxygen/5% carbon dioxide-equilibrated ACSF at room temperature (24–25 °C) for more than 1 h prior to their use for electrophysiological recordings.

### 4.4. Cerebellar Purkinje Cell Whole-Cell Recording, Biocytin Histochemistry and Parallel-Fiber Electrical Stimulation

Whole-cell patch-clamp recordings were performed on the somata of Purkinje cells (PCs) in cerebellar slices, which were visualized using a 60× water-immersion objective lens mounted on a Nikon microscope (Eclipse FN1, Nikon Corporation, Tokyo, Japan). The extracellular solution employed was magnesium-free ACSF, whose components (in mM) were as follows: 125 sodium chloride (NaCl), 3 potassium chloride, 2.5 calcium chloride, 1 sodium dihydrogen phosphate, 26 sodium bicarbonate, and 10 D-glucose (pH 7.4; osmotic pressure 295–305 mOsm). The components of the internal solution for patch-clamp recording electrodes (in mM) were as follows: 120 potassium gluconate, 10 HEPES, 1 EGTA, 5 potassium chloride, 3.5 cesium chloride, 4 sodium chloride, 8 biocytin, 4 disodium ATP, and 0.2 disodium GTP. The pH was adjusted to 7.3 with potassium hydroxide (KOH), and the osmolarity was set to 300 mOsm. The resistance of the recording electrode in the bath solution ranged from 4 to 6 MΩ, and the series resistance varied between 10 and 20 MΩ. Membrane potentials and/or currents were recorded using a patch-clamp amplifier (Axopatch 700B, Molecular Devices, Foster City, CA, USA), filtered at 5 kHz, and the data were acquired via a Digidata 1550 series analog-to-digital interface on a personal computer using Clampex 10.4 software (Molecular Devices, Foster City, CA, USA). To record PF–PC excitatory postsynaptic currents (EPSCs), the PCs were voltage-clamped at a holding potential of −70 mV. Series resistance was monitored by applying voltage pulses (10 ms, 5 mV), and only cells with stable series resistance were included in the analysis.

For PF electrical stimulation, a stimulating electrode containing ACSF (with a resistance of 0.1–0.5 MΩ) was placed in the molecular layer of the cerebellar slice. Paired current pulses (0.2, 10–100 μA, 50 ms) were delivered at a frequency of 0.5 Hz. The paired-pulse ratio (PPR) was calculated as the ratio of the amplitude of the second EPSCs (N2) to that of the first EPSCs (N1). In some experiments, protein kinase inhibitor-(6-22) amide (PKI) was added to the pipette internal solution to inhibit the activity of postsynaptic protein kinase A (PKA) [43]. During the recording of miniature excitatory postsynaptic currents (mEPSCs), gabazine (20 μmol/L) and tetrodotoxin (TTX; 1 μmol/L) were added respectively to block γ-aminobutyric acid (GABA)-ergic synaptic transmission and spontaneous EPSCs induced by excitatory inputs to PCs [43].

### 4.5. Immunohistochemistry, Confocal Fluorescence Imaging, and Co-Immunoprecipitation

Four mice were deeply anesthetized by intraperitoneal injection of 2,2,2-tribromoethanol (250 mg/kg). Subsequently, phosphate-buffered saline (PBS, pH 7.4) was perfused through the heart, followed by perfusion with 4% paraformaldehyde (PFA; Sinopharm Chemical Reagent Co., Ltd., Beijing, China). After the brain was fixed in 4% PFA at 4 °C for 48 h, it was washed with PBS every 10 min for a total of 3 washes. Thereafter, the brain tissue was dehydrated sequentially in PBS solutions containing 10%, 20%, and 30% sucrose. The brain tissue was embedded in Tissue-Tek O.C.T. embedding medium (Zhongshan Jinqiao Biotechnology Co., Ltd., Beijing, China) and then rapidly frozen at −80 °C for 2 h. Coronal brain sections with a thickness of 40 μm were cut using a Leica (Germany) CM3050S cryostat (Leica Biosystems Nussloch GmbH, Nussloch, Germany). The sections were mounted on glass slides, then permeabilized with PBS solution containing 0.3% Triton X-100, followed by blocking with PBS solution containing 10% donkey serum. Subsequently, mouse anti-GluN2A antibody (catalog number: SAB5200888; dilution ratio: 1:100, Merck, Shanghai, China) and rabbit anti-calcium channel (α2/δ-1 subunit) antibody (catalog number: C5105; dilution ratio: 1:50, Merck) were added and incubated overnight at 4 °C. After the incubation was completed, Alexa Fluor 488 donkey anti-mouse secondary antibody (catalog number: A21202; dilution ratio: 1:500, Thermo Fisher Scientific, Waltham, MA, USA), Cy5™ goat anti-rabbit IgG secondary antibody (catalog number: A10523; dilution ratio: 1:1000, Thermo Fisher Scientific, Waltham, MA, USA), and 4′,6-diamidino-2-phenylindole (DAPI; catalog number: D3571; dilution ratio: 1:1000, Thermo Fisher Scientific, Waltham, MA, USA) were added and incubated at room temperature for 2 h. After the sections were washed 3 times with PBS and slightly dried, they were mounted with anti-fade mounting medium. Fluorescence images were acquired using a Nikon (Tokyo, Japan) A1RMP high-speed multiphoton confocal laser-scanning microscope.

For the co-immunoprecipitation (Co-IP) assay, cerebellar cortical molecular layer tissues (n = 4 mice) were dissected and homogenized in ice-cold immunoprecipitation lysis buffer (#87788; Thermo Fisher Scientific), which contains 25 mmol Tris-HCl (pH 7.4), 150 mmol NaCl, 1% NP-40, 1 mmol EDTA, 5% glycerol, and a protease inhibitor cocktail. The homogenate was then incubated on ice for 30 min to prepare total protein. The lysates were centrifuged at 16,000× *g* for 15 min to collect the supernatants. Protein concentrations of the supernatants were subsequently determined. Thereafter, the samples were incubated overnight at 4 °C with Protein A + G Magnetic Beads (#P2108m, Beyotime, Shanghai, China) pre-conjugated with rabbit anti-GluN1 antibody (#G8913, 1:100 dilution; Sigma-Aldrich, St. Louis, MO, USA). Rabbit IgG-bound Protein A + G beads were used as negative controls. All samples were washed three times with immunoprecipitation buffer, followed by immunoblotting with mouse anti-α2δ-1 antibody (#sc-271697, 1:500 dilution; Santa Cruz Biotechnology, Santa Cruz, CA, USA). Protein bands were visualized using ECL Western Blotting Substrate (WBKLS0100, Millipore, Billerica, MA, USA).

### 4.6. Westen Blot Analysis

A total of 12 mice were used for Western blot analysis. Tissue samples from the cerebellar cortical molecular layer of each mouse were randomly assigned to three groups: control (ACSF), PGB, and α_2_δ-1 Tat peptide + PGB. Samples from each group were snap-frozen in liquid nitrogen and stored at −80 °C until Western blot analysis. Samples were homogenized in RIPA lysis buffer (Santa Cruz Biotechnology) supplemented with protease inhibitors for 15 min, followed by centrifugation at 14,000× *g* for 30 min at 4 °C. The supernatant was collected, and protein concentration was quantified using the detergent-compatible DC Protein Assay (Bio-Rad Laboratories, Hercules, CA, USA). Equal amounts of protein were loaded onto 10% SDS-PAGE gels for electrophoresis and then transferred onto nitrocellulose membranes. Membranes were blocked with 5% non-fat blocking-grade milk (Bio-Rad) at 37 °C for 2 h, and incubated overnight at 4 °C with the following primary antibodies: anti-phosphorylated protein kinase A (anti-p-PKA, 1:1000, Cell Signaling Technology, Danvers, MA, USA), anti-protein kinase A (anti-PKA, 1:1000, Cell Signaling Technology, Danvers, MA, USA), and anti-β-actin (1:5000, Santa Cruz Biotechnology, Dallas, TX, USA). Subsequently, membranes were incubated with appropriate peroxidase-conjugated secondary antibodies (1:5000, Santa Cruz Biotechnology, Dallas, TX, USA) at 37 °C for 1 h. Protein bands were visualized using the BeyoECL Plus chemiluminescence kit and an ECL detection system (Epizyme, Shanghai, China), followed by quantitative analysis with ImageJ software (version 1.8.0, National Institutes of Health, Bethesda, MD, USA).

### 4.7. Stereotaxic Surgery, Glutamate Sensor Viral Nano-Injection and Glutamate Sensor Fluorescence Imaging Analysis

Stereotaxic surgery, nano-injection of glutamate sensor-expressing virus, and fluorescence analysis were performed as previously described [42]. Briefly, 2–3-week-old mice (n = 4) were anesthetized with 4% isoflurane (R500, RWD Life Science, Shenzhen, Guangdong, China) and secured in a stereotaxic frame (68801, RWD, Shenzhen, China) under sterile conditions for craniotomy. Anesthesia was maintained with 1–2% isoflurane throughout the surgical procedure. Virus was loaded into glass micropipettes (tip diameter ~20 μm) fabricated using a micropipette puller (P97, Sutter Instrument, Novato, CA, USA), and injected via a Nanoject III micro-injector (Drummond Scientific, Broomall, PA, USA). A total volume of 300 nL of glutamate sensor virus (AAV2/9-hSyn-SF-iGluSnFR.A184S-WPRE, Obio Technology, Shanghai, China) was unilaterally injected into three sites of the cerebellar cortex with the following stereotaxic coordinates: anteroposterior (AP): −7.0 mm; mediolateral (ML): +3.02, +3.12, and +3.22 mm; dorsoventral (DV): −2.25 mm. The virus was infused at a rate of 1 nL/s, and the micropipette was kept in place for 10 min after injection to minimize back-flow. Mice were allowed to recover for at least 4 weeks post-injection to ensure sufficient sensor expression before slice recordings.

Four weeks later, AAV2/9-iGluSnFR.A184S-injected mice were used for cerebellar slice preparation. Cerebellar slices were transferred to a recording chamber mounted on an upright Nikon Eclipse FN1 microscope and continuously perfused with oxygenated ACSF. Whole-cell patch-clamp recordings were obtained from the somata of PCs, while iGluSnFR fluorescence imaging was performed simultaneously in cerebellar slices. Fluorescence signals were visualized using a 40× water-immersion objective (NIR Apo 40×/0.80 W DIC N2, WD 3.5 mm; Nikon). iGluSnFR.A184S was excited at 488 nm via a DG-4 wavelength-switching light source (Sutter Instrument, Novato, CA, USA). Fluorescence images were acquired at 15 Hz using a Hamamatsu ORCA-Flash4.0 LT digital CMOS camera controlled by HCImage software (Ver. 4.5.0, Hamamatsu Photonics, Hamamatsu, Japan). Each imaging acquisition epoch lasted 3 s, with an intertrial interval of 30 s. Fluorescence images were analyzed offline using ImageJ. Background fluorescence was subtracted from each frame using a region without tissue fluorescence. Regions of interest were selected in the molecular layer from the stimulation-evoked response area. Baseline fluorescence, F_0_, was defined as the mean fluorescence intensity during the −1 to 0 s prestimulation window. Fluorescence changes were calculated as ΔF/F = (F_t_ − F_0_)/F_0_ and expressed as percentages. where F_t_ represents the fluorescence measured at time point t, and F_0_ represents the mean fluorescence during the 1 s baseline period before stimulus application. Peak ΔF/F amplitude and area under the curve were used for quantitative analysis [42].

### 4.8. Chemical and Drug Application

Pregabalin (PGB), SR95531 (gabazine), D-APV (D-aminophosphonovaleric acid), PEAQX, KT5720, and PKA Inhibitor Fragment (6-22) amide TFA (PKI) were purchased from GLBIO (Montclair, NJ, USA), and tetrodotoxin (TTX) was obtained from Sigma-Aldrich (Shanghai, China). The α_2_δ-1 Tat peptide (VSGLNPSLWSIFGLQFILLWLVSGSRHYLW) was purchased from MedChemExpress LLC. (Shanghai, China). The stock solution of KT5720 (100 µM) was prepared in dimethyl sulfoxide (DMSO). Throughout all experiments, the final concentration of DMSO was less than 0.1%, which did not affect or induce any currents in separate control experiments. To avoid the effects of PGB on neurons and synaptic transmission outside the molecular layer, PGB was micro-applied to the middle region of the molecular layer above the recorded Purkinje cells (PCs) at a rate of 0.1 μL/s for 300 s using a micro-injection pump (KDS-210, KD Scientific, Holliston, MA, USA). All other chemicals were bath-applied to cerebellar slices in ACSF at a flow rate of 0.5 mL/min using a peristaltic pump (Gilson Minipulse 3; Villiers-le-Bel, France). In experiments involving KT5720, drug application was initiated at least 10 min before recording and maintained throughout the experimental period. Gabazine (20 µM) was included in the ACSF during all recordings to block GABAA receptor-mediated inhibitory synaptic responses.

After electrophysiological recordings, cerebellar slices were collected and fixed in 4% paraformaldehyde prepared in 0.1 M phosphate buffer (pH 7.4). Following overnight incubation with the avidin–biotin complex (ABC Elite Kit; Vector Laboratories, Burlingame, CA, USA) at room temperature, biocytin was visualized via 3,3′-diaminobenzidine tetrahydrochloride histochemistry.

### 4.9. Data Analysis

All experimental data were first subjected to post hoc power analysis using G*Power 3.1. Sample sizes (n) for all comparisons were determined with a statistical power > 0.8 at α = 0.05. Acquired electrophysiological data were analyzed using Clampfit 11.6 (Molecular Devices, Foster City, CA, USA). The paired-pulse ratio (PPR) was calculated as the amplitude ratio of the second EPSC (N2) to the first EPSC (N1). The frequency and amplitude of miniature excitatory postsynaptic currents (mEPSCs) were quantified with MiniAnalysis software (Version 6.0.3; Synaptosoft, Decatur, GA, USA). Raw mEPSC traces were digitally filtered at 1 kHz, and only synaptic events with well-defined baselines and peaks were included in amplitude analysis. For mEPSC detection, the threshold was set to 4 pA and the event search window to 30 ms. All parameters were kept constant for each recorded cell during ACSF perfusion, drug application, and washout. ImageJ software (version 1.8.0) was used for the densitometric analysis of PKA and p-PKA band gray values in Western blots. The normality of experimental data was examined using the Kolmogorov–Smirnov test, and one-way ANOVA repeated-measures with Tukey’s post hoc test (SPSS software; Version 26, Chicago, IL, USA) was used to determine the level of statistical significance between groups of data. P values below 0.05 were considered to indicate a statistically significant difference between experimental groups.

## 5. Conclusions

The pharmacological action of PGB is generally attributed to the inhibition of the α2δ-1 isoform of VGCCs. PGB binds to α_2_δ1 VGCCs and modulates the release of glutamate, GABA, and norepinephrine in the central nervous system [4,5,6,7]. Importantly, VGCCs containing the α_2_δ subunit are widely distributed throughout the cerebellar cortex, including P/Q-, N-, and R-type calcium channels. These channels regulate presynaptic neurotransmitter release onto PCs and modulate the structural and functional synaptic organization within the cerebellar cortex [33,34]. The present results demonstrated that GluN2A and α_2_δ-1 subunit immunoreactivity colocalized around the dendrites and somata of cerebellar PCs. Additionally, co-immunoprecipitation assays showed that α_2_δ-1 was co-precipitated with GluN1 in tissues from the molecular layer of the mouse cerebellum. Collectively, these findings suggest that α_2_δ-1–NMDAR complexes in the cerebellar cortex mediate the regulatory effect of PGB on PF–PC synaptic transmission.

PGB inhibited PF–PC EPSCs in a concentration-dependent manner, accompanied by an increase in the PPR. This effect was abolished by extracellular blockade of NMDA receptors, selective inhibition of the GluN2A subtype, or disruption of α_2_δ-1–NMDAR complexes. These findings indicate that PGB depresses PF–PC synaptic transmission by modulating presynaptic α_2_δ-1–NMDAR complexes in mouse cerebellar slices in vitro. Furthermore, PGB significantly reduced the fluorescence intensity of glutamate released upon PF stimulation and decreased the frequency of mEPSCs in cerebellar PCs. These inhibitory effects were completely eliminated by extracellular blockade of GluN2A-containing NMDA receptors or disruption of α_2_δ-1–NMDAR complexes, suggesting that the PGB-induced reduction in mEPSC frequency is mediated by α_2_δ-1/GluN2A-assembled NMDA receptor complexes. Moreover, the inhibitory effects of PGB on both PF–PC EPSCs and mEPSCs were abrogated by extracellular PKA inhibition, but not by intracellular PKA inhibition. Western blot analysis revealed that PGB increased PKA phosphorylation in the molecular layer of the mouse cerebellar cortex. These results confirm that PGB reduces presynaptic glutamate release through the presynaptic PKA signaling pathway. Collectively, the present findings demonstrate that PGB suppresses glutamate release from PF terminals in the mouse cerebellar cortex via the presynaptic α_2_δ-1 subunit-coupled GluN2A-containing NMDA receptor/PKA signaling cascade, thereby inhibiting PF–PC synaptic transmission. These results provide novel mechanistic insights into the central nervous system effects of PGB and suggest that PGB reshapes cerebellar PC output, which may further regulate motor coordination and motor learning in living animals.

## Data Availability

The original contributions presented in this study are included in the article. Further inquiries can be directed to the corresponding authors.
